# Does beer, wine or liquor consumption correlate with the risk of renal cell carcinoma? A dose-response meta-analysis of prospective cohort studies

**DOI:** 10.18632/oncotarget.3749

**Published:** 2015-04-29

**Authors:** Xin Xu, Yi Zhu, Xiangyi Zheng, Liping Xie

**Affiliations:** ^1^ Department of Urology, The First Affiliated Hospital, School of Medicine, Zhejiang University, Hangzhou, China

**Keywords:** alcohol, renal cell carcinoma, epidemiology, risk factor, meta-analysis

## Abstract

Despite plenty of evidence supports an inverse association between alcohol drinking and risk of renal cell carcinoma (RCC), sex-specific and beverage-specific dose-response relationships have not been well established. We examined this association by performing a systematic review and meta-analysis of prospective studies. Studies were identified by comprehensively searching PubMed and EMBASE databases through February 21, 2015. Categorical and dose-response meta-analyses were conducted to identify the effects of alcohol on RCC. A total of eight publications (including seven cohort studies and one pooled analysis of 12 cohort studies) were eligible for this meta-analysis. Dose-response analysis showed that each 5 g/day increment of alcohol intake corresponded to a 5% decrease in risk of RCC for males and 9% for females. Alcohol intakes from wine, beer, and liquor were each associated with a reduced risk of RCC. When these associations were examined separately by gender, statistically significant inverse associations were restricted to alcohol from wine among females (RR = 0.82, 95% CI 0.73–0.91) and to alcohol from beer and from liquor among males (RR = 0.87, 95% CI 0.83–0.91 and RR = 0.95, 95% CI 0.92–0.99, respectively). In conclusion, there exist gender-specific and beverage-specific differences in the association between alcohol intake and RCC risk.

## INTRODUCTION

Kidney cancer is one of the ten most common types of cancer worldwide, with over 271,000 newly diagnosed cases and over 116,000 deaths in 2008 [1]. The most common form, renal cell carcinoma (RCC) of the renal parenchyma, accounts for over 85% of all kidney cancers [2, 3]. RCC incidence rates vary substantially over the world. Rates are generally high in more developed regions (e.g., Europe and North America) and low in less developed areas (e.g., Asia and South America) [2]. RCC occurs twice as often in males as in females [4]. Although the reason for the higher incidence in developed countries and in males is not very clear [5], the nonhomogeneity of RCC incidence rates implies the existence of modifiable risk factors. On the other hand, RCC incidence is rising by approximately 2 to 3% per year [1, 6], which is due in large part to the increasing use of diagnostic imaging (ultrasound, magnetic resonance imaging, and computerized tomography scan) [7]. However, both advanced stage disease and the mortality rate for renal cancers have also been increasing, suggesting that risk factors have played some role in this upward trend [3, 6]. Based on the current evidence, cigarette smoking, obesity and hypertension are the most well established risk factors for RCC [2, 3].

Recent epidemiological studies have suggested that moderate alcohol drinking may be associated with a reduced risk of RCC [2, 3, 8]. Two well-done systematic reviews conducted in 2012 also supported this idea [9, 10]. Nevertheless, the results of recently published prospective cohort studies remain inconsistent or conflicting [11–13]. Karami et al. [12] showed that increasing alcohol consumption was associated with reduced RCC risk. Macleod et al. [11] found the trend, but they just did not reach the statistical significance. Washio et al. [13] reported that alcohol drinking was not related to the kidney cancer mortality. Moreover, sex-specific and beverage-specific (beer, wine or liquor) relationship between alcohol intake and RCC risk has not yet been established. We are wondering whether there is a gender difference in this association and whether different types of alcoholic beverages affect RCC risk differently.

We therefore conducted a dose-response meta-analysis on alcohol consumption and RCC risk by summarizing the results of all relevant prospective studies. Our aim was to update evidence on the association between them, and to quantify the sex-specific and beverage-specific dose-response relationships.

## RESULTS

### Literature search and study characteristics

**Figure 1:**
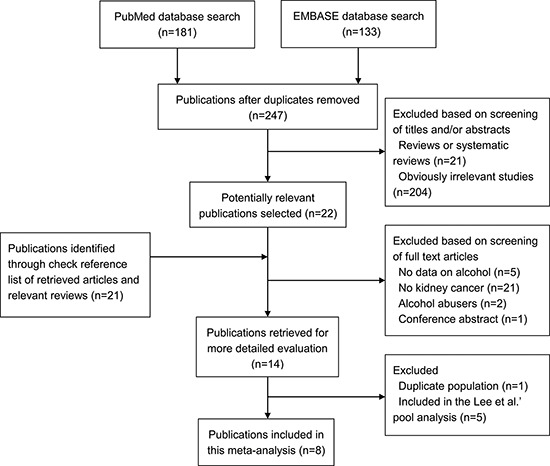
Flowchart of selection of studies for inclusion in this meta-analysis

We identified eight publications of alcohol drinking and RCC risk [11, 12, 14–19] (Figure 1), including seven independent cohort studies and one pooled analysis of 12 cohort studies. A total of 5,503 RCC cases were eligible for this systematic review. Four studies were conducted in United States [11, 12, 17, 19], one in Japan [16], one in Korea [18], one in United Kingdom [15], and one in multiple countries [14]. Studies were published between 2007 and 2014. The studies [11, 12, 14–19] that provided at least 3 quantitative categories of alcohol drinking were included in dose-response meta-analysis. Most studies adjusted for age [11, 14–19], race [11, 12, 17, 19], smoking [11, 12, 14, 15, 17–19], body mass index (BMI) [11, 12, 14, 15, 18, 19], hypertension [11, 12, 14, 17–19], and physical activity [15, 17–19]. Six studies [11, 12, 14, 15, 17, 19] used incidence of renal cell carcinoma as outcome, and two [16, 18] used kidney cancer mortality. The points of study quality assessed by Newcastle-Ottawa quality assessment scale ranged from 6 to 9 (with a mean of 7.5). Detailed characteristics of the included studies are presented in Table 1.

**Table 1:** Main characteristics of studies included in this meta-analysis

First author, year, country	Gender (age)	Alcohol assessment	Outcome	No. of cases	No. of cohort	Duration of follow-up	Quality score	Adjustment variables
Karami et al., 2014, USA	MF (55–74, range)	Questionnaire	Incidence of renal cell carcinoma	408	107,998 (PR)	11.4 y	7	Sex, race, hypertension, BMI, smoking status, and study center
Macleod et al., 2013, USA	MF (50–76, range)	FFQ	Incidence of renal cell carcinoma	249	77,260 (PR)	8 y	8	Age, gender, race, BMI, smoking, fruit intake, vegetable intake, hypertension, diabetes, kidney disease, and viral hepatitis
Lew et al., 2011, USA	M (50–71, range)	FFQ	Incidence of renal cell cancer	1,348	293,466 (PR)	8 y	8	Age, race, education, marital status, BMI, smoking, physical activity, hypertension, and intakes of protein and total energy excluding calories from alcohol.
Lew et al., 2011, USA	F (50–71, range)	FFQ	Incidence of renal cell cancer	466	198,721 (PR)	8 y	8	Age, race, education, marital status, BMI, smoking, physical activity, hypertension, and intakes of protein and total energy excluding calories from alcohol.
Kim, et al., 2010, Korea	M (40–69, range)	Health examination	Kidney cancer mortality	74	919,199 (PR)	5 y	7	Age, residential, smoking status, ≥3 times/week regular exercise, BMI, systolic and diastolic blood pressure, and fasting blood sugar
Allen et al., 2009, UK	F (55, average)	Questionnaire	Incidence of renal cell carcinoma	1,141	1,280,296 (PR)	7.2 y	7	Age, region of residence, socioeconomic status, BMI, smoking, physical activity, use of oral contraceptives, and hormone replacement therapy
Setiawan et al., 2007, USA	M (59.3, mean)	Questionnaire	Incidence of renal cell cancer	220	75,162 (PR)	8.3 y	8	Age, ethnicity, smoking, hypertension, and physical activity
Setiawan et al., 2007, USA	F (58.8, mean)	Questionnaire	Incidence of renal cell cancer	127	85,964 (PR)	8.3 y	8	Age, ethnicity, smoking, hypertension, andphysical activity
Ozasa et al., 2007, Japan	M	NA	Kidney cancer mortality	28	427,155 (PY)	NA	6	Age and area of study
Ozasa et al., 2007, Japan	F	NA	Kidney cancer mortality	12	642,381(PY)	NA	6	Age and area of study
Lee et al., 2007, International	MF (15–107, range)	FFQ	Incidence of renal cell cancer	1,430	760,044(PR)	7–20 y	9	Age, history of hypertension, BMI, pack-years of smoking, combination of parity and age at first birth, and total energy intake

BMI, body mass index; No., number; MF, male and female; F, female; M, male; y, years; FFQ, food frequency questionnaire; PR, person at risk; PY, person year; NA, not available

### Categorical meta-analysis

Supplementary Figure S1 shows the study-specific and pooled RRs and 95% CIs of RCC for any, light, moderate, and heavy drinking. When compared with non/occasional drinking, the pooled RRs were 0.86 (95% CI 0.76–0.96; *I^2^* = 60.1%) for any, 0.92 (95% CI 0.83–1.01; *I^2^* = 45.2%) for light, 0.75 for (95% CI 0.66–0.86; *I^2^* = 45.1%) for moderate, and 1.08 (95% CI 0.42–2.75; *I^2^* = 74.8%) for heavy drinking.

Table 2 gives the pooled RRs and 95% CIs of RCC at different levels of alcohol drinking in strata of relevant factors. For any drinking, significant results were found in subgroups of females (RR = 0.84, 95% CI 0.75–0.94; *I^2^* = 24.6%), USA (RR = 0.87, 95% CI 0.76–0.99; *I^2^* = 51.3%), quality score ≥ mean (RR = 0.89, 95% CI 0.79–0.99; *I^2^* = 71.7%), Caucasian (RR = 0.89, 95% CI 0.81–0.97; *I^2^* = 44.0%), and incidence (RR = 0.88, 95% CI 0.81–0.95; *I^2^* = 34.7%). For light drinking, results were not materially different in strata of area, ethnicity, and outcome, but not in other strata. For moderate drinking, results were basically consistent in strata of sex and major confounders adjusted, but not in other strata.

**Table 2:** Pooled and subgroup analyses stratified by sex, area, major confounders adjusted, quality score, ethnicity, and outcome

	Any versus non/occasional				Light versus non/occasional				Moderate versus non/occasional			
	*N*^a^	RR (95% CI)	*I^2^*(%)	*P* value^b^	*N*^a^	RR (95% CI)	*I^2^*(%)	*P* value^b^	*N*^a^	RR (95% CI)	*I^2^*(%)	*P* value^b^
Overall	8	0.86 (0.76–0.96)	60.1	0.005	6	0.92 (0.83–1.01)	45.2	0.078	8	0.75 (0.66–0.86)	45.1	0.068
Sex^c^												
Male	6	0.86 (0.70–1.06)	70.9	0.004	5	0.99 (0.85–1.16)	43.2	0.134	6	0.76 (0.62–0.92)	46.7	0.095
Female	6	0.84 (0.75–0.94)	24.6	0.249	5	0.87 (0.80–0.95)	0.0	0.840	4	0.72 (0.62–0.84)	6.7	0.360
Area^d^												
USA	4	0.87 (0.76–0.99)	51.3	0.086	3	0.93 (0.79–1.09)	57.2	0.053	4	0.75 (0.63–0.90)	46.9	0.110
Europe	1	0.86 (0.75–0.98)	-	-	1	0.89 (0.77–1.02)	-	-	1	0.78 (0.66–0.93)	-	-
Asia	2	1.91 (0.35–10.48)	84.6	0.001	1	0.63 (0.35–1.13)	-	-	2	1.07 (0.18–6.41)	84.3	0.012
Major confounders adjusted^e^												
Yes	4	0.86 (0.73–1.02)	70.8	0.008	3	0.95 (0.80–1.13)	68.9	0.022	4	0.75 (0.62–0.90)	55.1	0.063
No	4	0.84 (0.70–1.02)	49.8	0.077	3	0.87 (0.78–0.97)	0	0.914	4	0.75 (0.60–0.94)	45.7	0.137
Quality score												
≥Mean	4	0.89 (0.79–0.99)	71.7	0.007	3	0.94 (0.82–1.08)	58.4	0.047	4	0.76 (0.66–0.89)	44.6	0.124
<Mean	4	0.84 (0.61–1.16)	47.2	0.092	3	0.87 (0.78–0.98)	0	0.526	4	0.74 (0.54–1.01)	58.1	0.067
Ethnicity^f^												
Caucasian	5	0.89 (0.81–0.97)	44.0	0.112	4	0.95 (0.85–1.06)	57.7	0.051	5	0.77 (0.68–0.86)	35.5	0.170
Asian	2	1.91 (0.35–10.48)	84.6	0.001	1	0.63 (0.35–1.13)	-	-	2	1.07 (0.18–6.41)	84.3	0.012
Outcome												
Incidence	6	0.88 (0.81–0.95)	34.7	0.151	5	0.93 (0.84–1.02)	45.4	0.089	6	0.76 (0.69–0.85)	26.5	0.226
Mortality	2	1.91 (0.35–10.48)	84.6	0.001	1	0.63 (0.35–1.13)	-	-	2	1.07 (0.18–6.41)	84.3	0.012

a The number of studies included.

b *P* for heterogeneity in subgroups.

c Studies which reported or could calculate the sex-specific estimates were selected.

d Study reported by Lee et al. containing multiple countries was not included in the subgroup analysis of area.

e Studies adjusted for age, smoking, body mass index, and hypertension.

f Study reported by Setiawan et al. containing multiple ethnicities was not included in the subgroup analysis of ethnicity.

Supplementary Figure S2 presents the results of sensitivity analysis for any, light, and moderate drinking. The pooled RRs with 95% CIs ranged from 0.82 (95% CI 0.74–0.92) to 0.88 (95% CI 0.79–0.98) for any, from 0.88 (95% CI 0.82–0.95) to 0.93 (95% CI 0.84–1.02) for light, and from 0.73 (95% CI 0.63–0.84) to 0.77 (95% CI 0.68–0.88) for moderate drinking, which indicated that the pooled estimates were robust and not influenced by a single study for any and moderate drinking, but not for light drinking. No significant publication bias was detected by Egger's test and Begg's test for any, light, and moderate drinking (All *P* > 0.5).

### Dose-response meta-analysis

**Figure 2:**
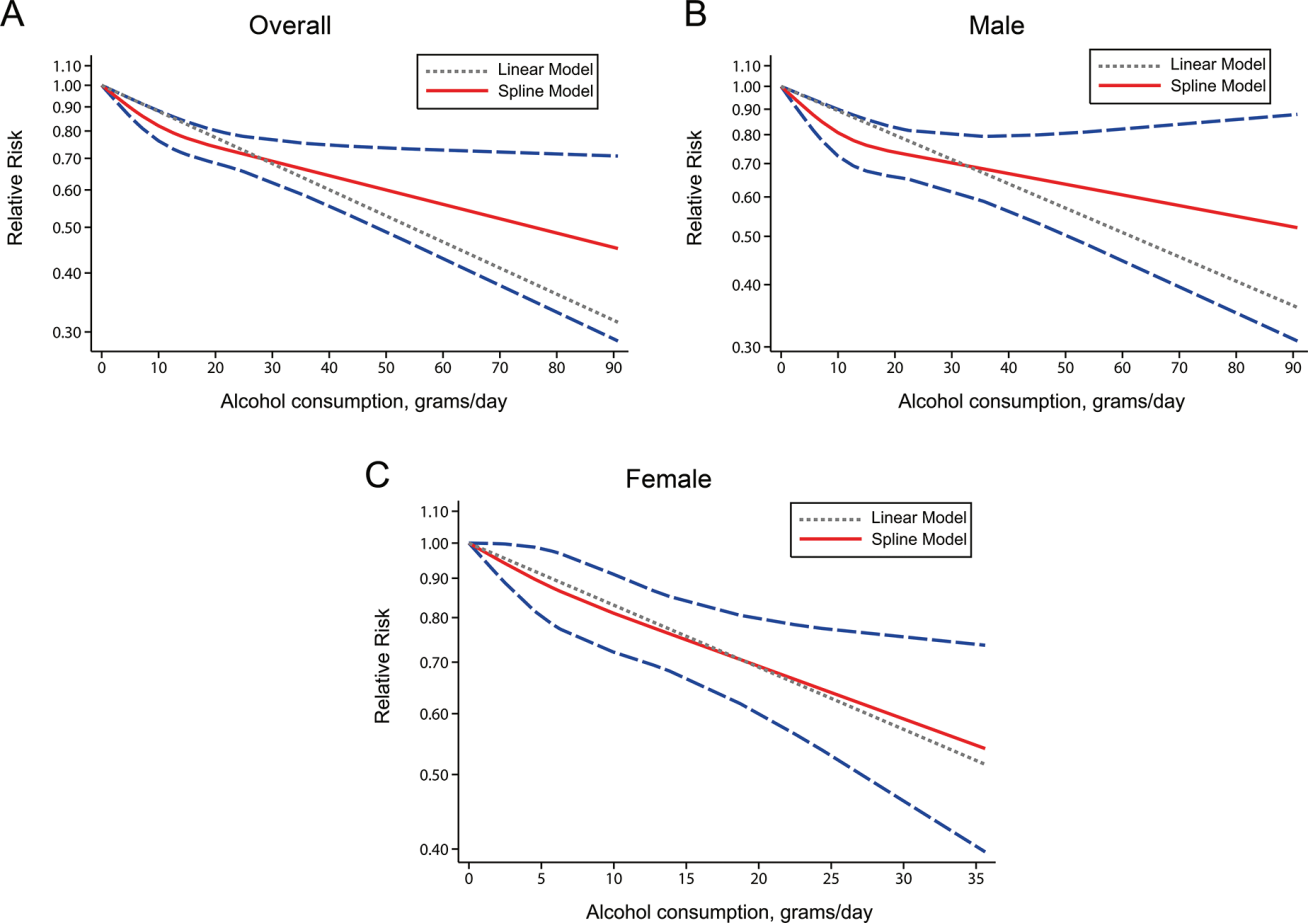
Relative risks (RRs) and the corresponding 95% confidence intervals (CIs) for the dose-response relationship between alcohol drinking (grams per day) and renal cell carcinoma (RCC) risk among the overall population **A.** males **B.** and females **C.** The solid line and the long dash line represent the estimated RRs and their 95% CIs. Short dash line represents the linear relationship.

The number of studies eligible for the dose-response analysis was eight [11, 12, 14–19], six [12, 14, 16–19], and five [12, 14, 15, 17, 19] for overall population, males, and females, respectively. Accordingly, the pooled RRs of RCC for 5 g/day increment of alcohol drinking decreased by 6% (RR = 0.94, 95% CI 0.92–0.95), 5% (RR = 0.95, 95% CI 0.93–0.97), and 9% (RR = 0.91, 95% CI 0.88–0.94), respectively. Some evidence of a nonlinear relationship between alcohol and RCC risk was found for overall population (*P* = 0.03 for nonlinearity) and males (*P* = 0.05 for nonlinearity). The downward trend was most obvious when alcohol intake increased up to about 12.5 g/day (Figure 2).

**Figure 3:**
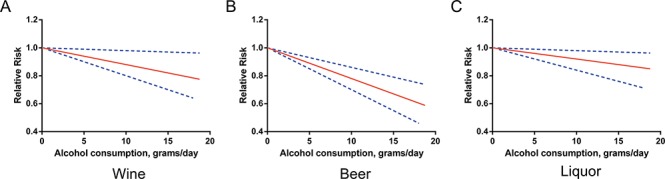
Relative risks (RRs) and the corresponding 95% confidence intervals (CIs) for the beverage-specific dose-response relationship in overall population The solid line and the long dash line represent the estimated RRs and their 95% CIs.

Three studies [12, 14, 19] were eligible for the beverage-specific dose-response analysis. Alcohol intakes from wine, beer, and liquor were each associated with a reduced risk of RCC among the overall population. The pooled RRs for a 5 g/day increase in alcohol intake were 0.94 (95% CI 0.90–0.99), 0.89 (95% CI 0.85–0.93), and 0.96 (95% CI 0.92–0.99), respectively (Figure 3). There was no evidence of nonlinearity. All *P* values for nonlinear assessment were >0.05.

**Figure 4:**
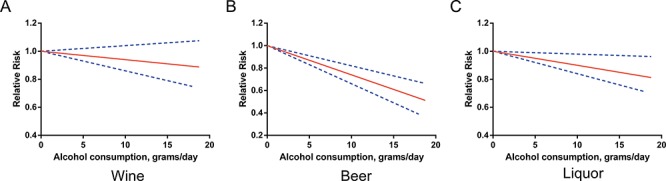
Relative risks (RRs) and the corresponding 95% confidence intervals (CIs) for the beverage-specific dose-response relationship in males The solid line and the long dash line represent the estimated RRs and their 95% CIs.

**Figure 5:**
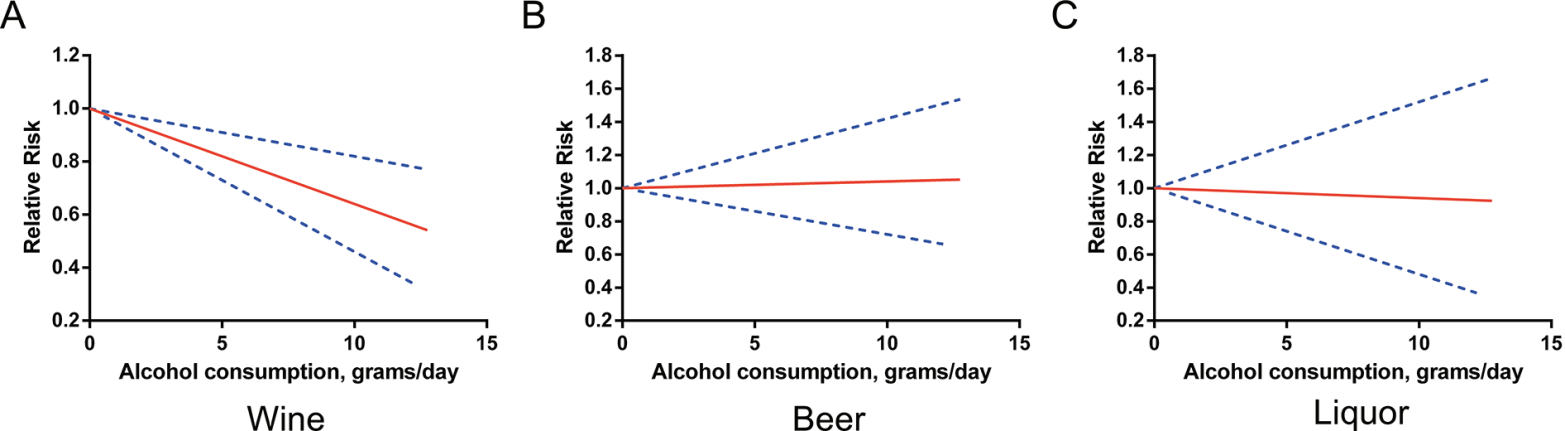
Relative risks (RRs) and the corresponding 95% confidence intervals (CIs) for the beverage-specific dose-response relationship in females The solid line and the long dash line represent the estimated RRs and their 95% CIs.

These beverage-specific associations were then examined separately by gender, and the statistically significant inverse associations were restricted to alcohol from beer and from liquor among males (RR = 0.87, 95% CI 0.83–0.91 and RR = 0.95, 95% CI 0.92–0.99, respectively) (Figure 4) and to alcohol from wine among females (RR = 0.82, 95% CI 0.73–0.91) (Figure 5). There was no evidence of nonlinearity. All *P* values for nonlinear assessment were >0.05.

In dose-response analysis, evidence of heterogeneity across studies was examined by the heterogeneity test. We did not find any significant difference among study-specific slopes in any of the dose-risk curve-fit model (All *P* > 0.10).

## DISCUSSION

The present meta-analysis summarized the evidence from all available prospective cohort studies and found a significant 25% decreased risk of RCC for moderate drinking (2–3 drinks/day), compared with non/occasional drinking. A slightly more beneficial effect was observed for females. The dose-response analysis showed that each 5 g/day increment of alcohol intake corresponded to a 5% decrease in risk of RCC for males and 9% for females. In beverage-specific dose-response analysis, alcohol intakes from wine, beer, and liquor were each associated with a reduced risk of RCC among the overall population. When beverage-specific associations were further examined separately by gender, statistically significant inverse associations were restricted to alcohol from beer and from liquor among males and to alcohol from wine among females. The findings of our study provide new and more detailed information for the inverse association between alcohol intake and RCC risk.

Several potential biologic mechanisms have been proposed to explain the inverse association between alcohol intake and RCC risk. First, light to moderate alcohol drinking is associated with improved insulin sensitivity [20, 21] and protective for type 2 diabetes [22]. Increased risk of RCC has been observed for individuals with diabetes or obesity [23, 24]. Second, alcohol contains antioxidant phenolic compounds, which can reduce oxidative stress and contribute to apoptosis by arresting cell cycle [25, 26]. Third, alcohol consumption has been inversely associated with the risk of chronic renal failure [27], which is an important risk factor for RCC development [28]. Forth, the diuretic effect of alcohol may also play a role by reducing the time that carcinogenic solutes are in contact with renal epithelial cells [14] and controlling hypertension [29], which is also a well-known RCC risk factor [2]. However, the association of total fluid intake with RCC risk has not been established [29, 30].

There are risk differences between the genders for total alcohol intake and for certain types of alcoholic beverage. Statistically significant inverse associations were restricted to alcohol from wine among females and to alcohol from beer and from liquor among males, which might be due to the discrepancy in the genetics, total body water, metabolic characteristics of alcohol, or other unknown factors [31, 32]. The most apparent relationships were observed for beer among males and for wine among females in this study, which were consist with the results of Lee et al.' pooled analysis [14] and another large prospective cohort study conducted by Lew et al. [19]. This may be partially because beer was the most popular beverage among males, while females mostly preferred wine [33]. Nevertheless, the exact mechanism underling the gender-specific and beverage-specific differences remains elusive and further investigation is warranted.

Two well-done systematic reviews performed by Bellocco et al. [9] and Song et al. [10] in 2012 has verified the inverse association of alcohol drinking with RCC risk. But their conclusions were drawn mainly based on case–control studies. Moreover, the results of recently published prospective cohort studies remain controversial [11–13]. Hence, an updated meta-analysis of prospective studies is needed and will provide more robust evidence as prospective studies can minimize selection biases and recall biases. In the present meta-analysis, despite of the limited number of included publications, the total sample size is large (more than 5,500 RCC cases). Furthermore, Lee et al.'s article [14] is consist of 12 cohort studies, thus a total of 19 independent cohort studies were actually analyzed in this meta-analysis. Apart from an inverse, non-linear association between total alcohol intake and RCC risk that has been suggested in previous meta-analyses, our study uncovers several novel and interesting findings on gender-specific and beverage-specific differences, which will provide some advice for people on choosing the appropriate drink (beer, wine, or liquor).

There are also great deals of research that have investigated the association of alcohol drinking with other site-specific cancer risk. According to a recent comprehensive review [34], RRs for heavy drinking compared with non/occasional drinking were 5.13 for oral and pharyngeal, 4.95 for esophageal, 2.65 for laryngeal, 2.64 for gallbladder, 2.07 for liver, 1.61 for breast, and 1.44 for colorectal cancer. Although our study found no association between heavy alcohol drinking and RCC risk, a large cohort study of alcohol abusers indicated a significantly elevated occurrence of renal cancer in this population [35]. In addition, although controversy still exists, emerging evidence has indicated that moderate drinkers tend to have better health and live longer than those who are either abstainers or heavy drinkers [36]. Moderate drinking has shown benefits on type 2 diabetes [22], heart health [37], strokes [38], Alzheimer's Disease [39], and so on. Our study offers some evidence to support the recommendation of moderate drinking, especially beer in males and wine in females. Klatsky et al.' study [40] assessed the role of alcoholic beverage choice in coronary risk and they also suggested that beer in males and wine in females had the strongest protection.

Our study has several strengths. First, all included studies were prospective cohort studies with a total of 5,503 RCC cases. Prospective studies have the advantage of being less subject to selection and recall bias than case–control studies. Second, the combined use of categorical and dose-response analyses provided a comprehensive assessment of the association between alcohol drinking and RCC risk. Third, we were able to evaluate the associations for males and females separately and for each alcoholic beverage.

Several limitations should also be discussed. First, significant heterogeneity was detected in categorical analysis. Therefore, these combined estimates should be treated with caution, although random-effects models were used to take heterogeneity into account. But no obvious heterogeneity was observed for dose-response analysis. Second, only published studies were included in our meta-analysis. Even if Begg's test and Egger' test did not suggest any evidence of publication bias, some inevitable publication bias may exist as studies with null results tend not to be published. Third, a meta-analysis is unable to solve problems with confounding factors that could be inherent in the included studies. The possibility of residual or unmeasured confounding cannot be excluded. Nevertheless, in majority of the original studies, main confounders (e.g., age, race, smoking, BMI, hypertension, and physical activity) were adjusted, or at least mostly. Finally, the number of studies included in the meta-analysis was limited. Because of this, more future prospective studies are warranted to confirm the conclusions in this study.

## CONCLUSION

This updated dose-response meta-analysis of prospective cohort studies suggests there exist gender-specific and beverage-specific differences in the association between alcohol intake and RCC risk. Statistically significant inverse associations were restricted to alcohol from beer and from liquor among males and to alcohol from wine among females. Future research is needed to investigate the potential mechanisms underlying these associations.

## MATERIALS AND METHODS

### Literature search

This systematic review and meta-analysis was performed according to meta-analysis of observation studies in epidemiology (MOOSE) guidelines [41]. A systematic literature search in PubMed and EMBASE databases from their inception to February 21, 2015 was conducted by two reviewers (XX and YZ), who understood about basic structure and functions of bibliographic and specialized databases, as well as the methodological and technical issues associated with searching [42]. We used the following search string in PubMed: (“kidney cancer” OR “kidney neoplasm” OR “kidney carcinoma” OR “renal carcinoma” OR “renal cancer” OR “renal cell cancer” OR “renal cell carcinoma”) AND (alcohol OR alcohols OR alcoholic OR ethanol OR wine OR beer OR liquor) AND (cohort OR prospective OR “nested case-control”). A similar strategy was used in EMBASE search. No language restriction was applied. We also reviewed the cited references from retrieved articles and reviews for additional relevant studies. In addition, we searched the grey literature extensively to check for publication bias. If relevant studies did not provide sufficient data for meta-analysis, we contacted authors to request missing data.

### Eligibility criteria

Articles included in this meta-analysis had to meet the following criteria: (*i*) cohort or nested case–control study conducted on the general population; (*ii*) one of the exposures was alcohol drinking; (*iii*) one of the outcomes was RCC risk; and (*iv*) studies reported risk estimates with their 95% confidence intervals (CIs), or data to calculate them. Studies on special populations (e.g., cohorts of alcoholics) [35, 43] were not included. If multiple publications from the same or overlapping population were available, the most informative one was included. In particular, we included a pooled analysis of 12 cohort studies [14], rather than separate publications, since seven of these cohorts had not previously reported on this association.

### Data extraction and quality assessment

Two authors independently extracted the following data in a standard format: first author's surname, publication year, country, gender, age, duration of follow-up, the method of exposure assessment, the number of cases, sample size (persons or person-years), covariates adjusted in the analysis, alcohol exposure levels and corresponding estimates with 95% CIs. For studies that reported several multivariable-adjusted relative risks (RRs), we extracted the RR estimate that was maximally adjusted for potential covariates to reduce the risk of possible residual confounding.

Two reviewers independently completed the quality assessment using the Newcastle–Ottawa Quality Assessment Scale (http://www.ohri.ca/programs/clinical_epidemiology/oxford.asp). This scale is an eight-item instrument used to assess the selection of study population, study comparability, and ascertainment of the outcome. The total score ranges from zero to nine, and higher scores indicate better methodological quality. Discrepancies were resolved by consensus and discussion.

### Statistical methods

We converted the amount of alcohol intake into a uniform measurement of grams (g) of ethanol per day by using the following equivalencies: 1 ml of alcohol as 0.8 g of ethanol, one drink as 12.5 g, and 1 ounce as 28 g [44, 45], if not otherwise specified in the original article. When a range of alcohol consumption was provided, the median or mean value was regarded as the corresponding exposure dose. If the median or mean value was not reported, we used the midpoint of each category. For upper, open-ended exposure categories, the exposure dose was defined by the lower bound added to the three-quarters of the adjacent previous category [46]. The lowest categories of exposure (i.e., non/occasional drinking) were treated as the reference group. Alcohol drinking were classified into three levels as light, moderate, and heavy drinking, which were defined as ethanol intake of <12.5 g/day (<1 drink/day), 12.5–37.5 g/day (2–3 drinks/day), and >37.5 g/day (>3 drinks/day), respectively. When multiple exposure categories lay in one of these levels, we pooled the corresponding estimates using the method proposed by Hamling et al. [47], thus taking into account their correlation. In the Million Women Study [15], we back calculate conventional 95% CIs from a set of RRs reported with 95% floated CIs [48].

We calculated pooled RRs using random effects model [49] that accounts for both within- and between-study heterogeneity, which was also assessed by the Q statistic (significance level at *P* < 0.10) and the *I^2^* score [50]. To evaluate the robustness of the pooled estimates, sensitivity analysis was performed by removing each study in turn. In order to investigate possible sources of heterogeneity among studies, we also conducted subgroups analyses by the available characteristics of studies and participants.

Dose-response meta-analysis was performed by the method described by Greenland et al. [51] and Orsini et al [52]. Only studies that considered at least 3 quantitative categories and provided the number of cases and person-years in each category were included. If studies only provided the overall person-years, we estimated the distribution of person-years using the method proposed by Aune et al. [53]. And if the overall person-years were also not reported, it was approximated from follow-up duration and number of individuals [54]. We examined a potential nonlinear dose-response relationship between alcohol intake and RCC risk using restricted cubic splines with three knots at the 25th, 50th, and 75th percentiles of the distribution. A *P* value for nonlinearity was calculated by testing the null hypothesis that the coefficient of the second spline was equal to zero [55].

Publication bias was evaluated by Begg's test (rank correlation method) [56] and Egger's test (linear regression method) [57]. All statistical analyses were performed with STATA 11.0 (StataCorp, College Station, TX) and a two-sided *P* < 0.05 was considered significant.

## SUPPLEMENTARY FIGURES


