# Supplementary material for: Does beer, wine or liquor consumption correlate with the risk of renal cell carcinoma? A dose-response meta-analysis of prospective cohort studies
Source: Oncotarget. 2015 Apr 29;6(15):13347–58. doi: 10.18632/oncotarget.3749 (PMC4537019; doi:10.18632/oncotarget.3749)
Supplement: Supplementary file 1 [file oncotarget-06-13347-s001.pdf]

SUPPLEMENTARY FIGURES

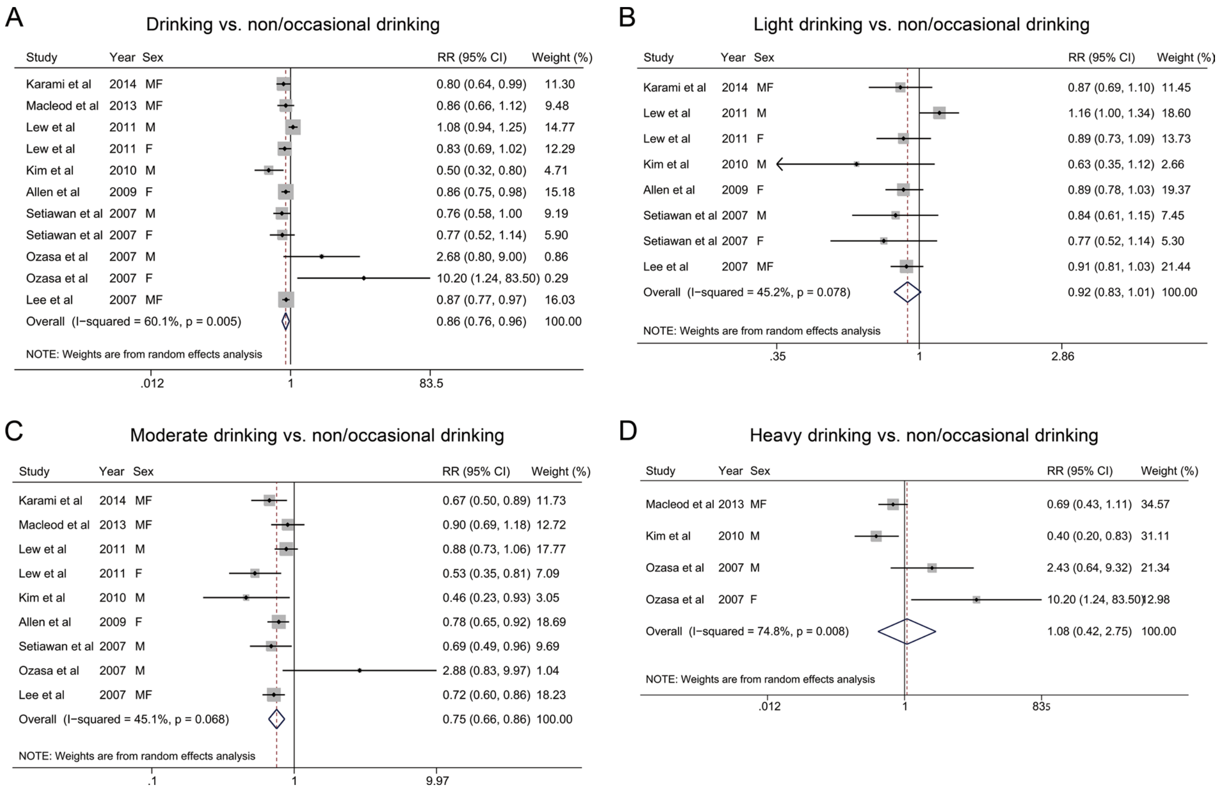

Supplementary Figure S1: Forest plots for pooled relative risks (RRs) and the corresponding 95% confidence intervals (CIs) of renal cell carcinoma (RCC) risk for any A. light B. moderate C. and heavy D. drinking.

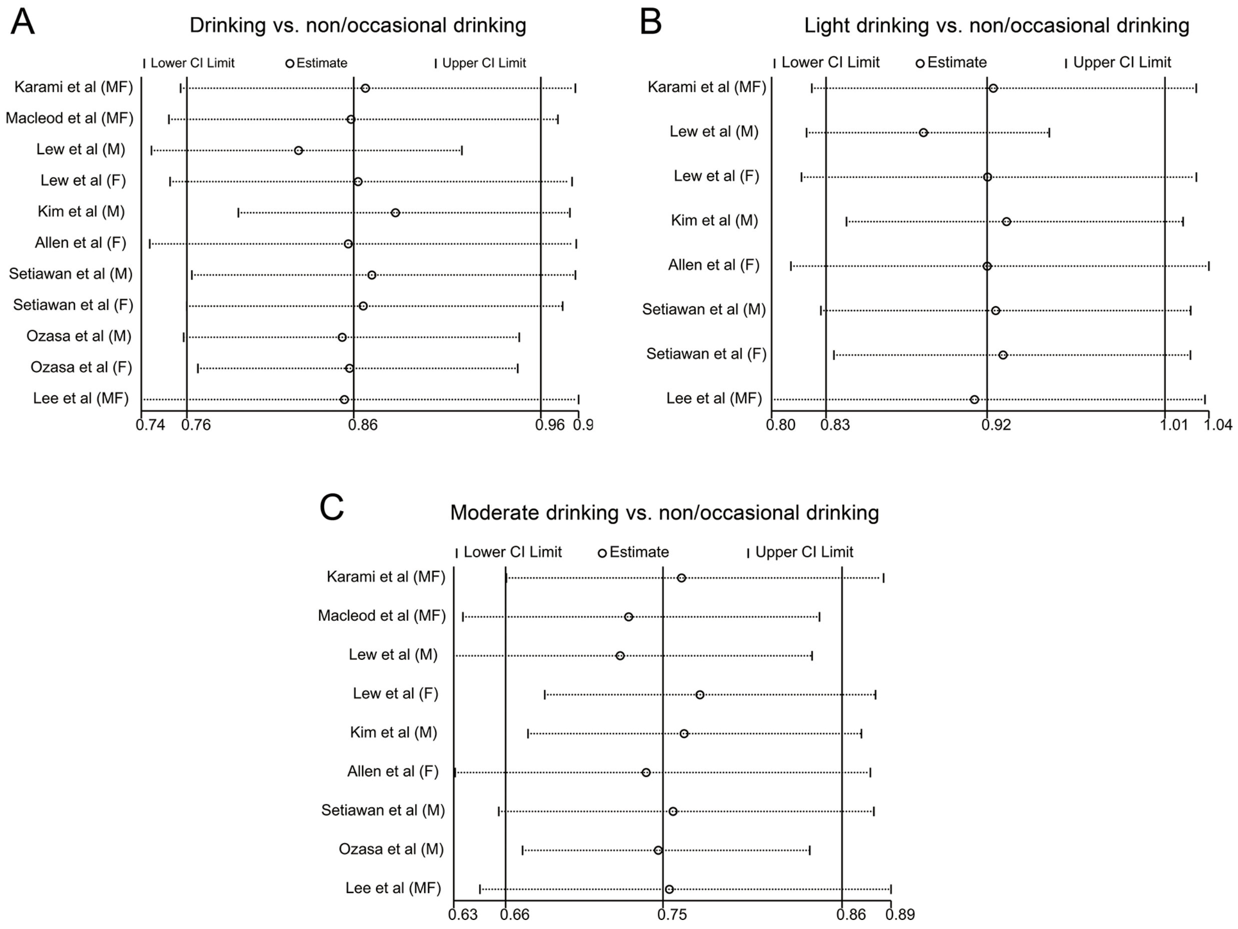

Supplementary Figure S2: Sensitivity analysis for any A. light B. and moderate C. drinking. Given name study is omitted.
